# Cross-sectional survey of selected enteric viruses in Polish turkey flocks between 2008 and 2011

**DOI:** 10.1186/s12917-017-1013-8

**Published:** 2017-04-14

**Authors:** K. Domańska-Blicharz, Ł. Bocian, A. Lisowska, A. Jacukowicz, A. Pikuła, Z. Minta

**Affiliations:** 1 Department of Poultry Diseases, National Veterinary Research Institute, Al. Partyzantów 57, 24-100 Puławy, Poland; 2 Department of Epidemiology and Risk Assessment, National Veterinary Research Institute, Al. Partyzantów 57, 24-100 Puławy, Poland

**Keywords:** Enteritis, Turkey, Astrovirus, Coronavirus, Rotavirus, Parvovirus, Statistics, Poland

## Abstract

**Background:**

Enteric diseases are an important health problem for the intensive poultry industry, resulting in considerable economic losses. Apart from such microbiological agents associated with enteritis as bacteria and parasites, a lot of research has been recently conducted on viral origin of enteric diseases. However, enteric viruses have been identified in intestinal tract of not only diseased but also healthy poultry, so their role in enteritis is still unclear. The present study aimed at determination of the prevalence of four enteric viruses, namely astrovirus, coronavirus, parvovirus and rotavirus in meat-type turkey flocks in Poland as well as at statistical evaluation of the occurrence of the studied viruses and their relationships with the health status and the age of birds. Two hundred and seven flocks of birds aged 1-20 weeks originating from different regions of the country were investigated between 2008 and 2011. Clinical samples (10 individual faecal swabs/flock) were duly processed and examined using molecular methods targeting the conservative regions of viral genomes: RNA-dependent RNA polymerase gene of astrovirus, non-structural 1 gene of parvovirus, non-structural protein 4 gene of rotavirus, and 5′ untranslated region fragment of turkey coronavirus. Different statistical methods (i.e. the independence chi-square test, the correspondence analysis and the logistic regression model) were used to establish any relationships between the analyzed data.

**Results:**

Overall, 137 (66.2%, 95% CI: 59.3-72.6) of the 207 turkey flocks sampled were infected with one or more enteric viruses. Among the 137 flocks, 74 (54%, 95% CI: 45.3-62.6) were positive for one virus, whereas 54 (39.4%, 9 5% CI: 31.2-48.1) and 9 (6.6%, 95% CI: 3.1-12.1) were co-infected with two or three different enteric viruses, respectively. No flock was simultaneously infected with all four viruses studied. The prevalence of astrovirus infection was 44.9% (95% CI: 38.0-52.0), parvovirus 27.5% (95% CI: 21.6-34.2), rotavirus 18.8% (95% CI: 13.8-24.8), and coronavirus 9.7% (95% CI: 6.0-14.5). Young turkeys aged 1-4 weeks old had the highest (82.1%, 95% CI:71.7-89.8) prevalence of viral infection. Applied statistical methods have indicated the dependence of rotavirus infection as well as the co-infection with multiple viruses and the health status of turkeys. Furthermore, our results statistically confirm that especially young birds are susceptible to infection with rotavirus and astrovirus.

**Conclusions:**

The study demonstrated the presence of astrovirus, coronavirus, parvovirus and rotavirus infections in Polish turkey farms. These viruses were detected in both healthy and diseased birds. However, the presented results provide valuable feedback which could help to evaluate the role of some enteric viruses in the etiology of enteritis in turkey.

## Background

Enteric diseases are an important health problem for the intensive poultry industry and can result in considerable economic losses. The most characteristic of these conditions are diarrhea, stunting, huddling, increased feed conversion and extended time-to-market. In the more severe forms, immune dysfunction and increased mortality may occur. Different terms have been used to describe enteric disease syndrome in turkey: poult enteritis complex (PEC) and poult enteritis mortality syndrome (PEMS) or light turkey syndrome (LTS) [1–3]. However, none of these descriptions relate to any specific agents, and numerous factors have been associated with them, including environmental, such as housing, ventilation, temperature and humidity; management, such as biosecurity programmes; and microbiological agents (viruses, bacteria and parasites). In recent years there has been a lot of research regarding the viral origin of enteric diseases, and different viruses, such as astrovirus, coronavirus, reovirus, rotavirus, parvovirus, and adenovirus have been identified in intestinal tract of not only diseased but also healthy poultry. The role of some viruses in enteritis is well-defined, i.e. coronavirus is a known factor of mud fever/bluecomb disease of turkeys [4]. However, the clinical and epidemiological significance of turkey infection with most of them still remains unclear. It seems that viruses are present in large amounts and different combinations in bird intestines, but in optimal nutritional and environmental conditions they do not cause clinical disease. In favorable conditions, such as feed change, sudden temperature shift or other stress factors, the viruses present in the gut might damage its walls making birds more susceptible to secondary bacterial infection. Furthermore, it is also possible that enteric viruses could contribute to the development of symptoms only by co-infections with multiple viruses. For a better understanding and control of enteric disease in turkeys, more studies on enteric viruses are needed.

In Polish commercial turkey flocks, despite the high level of hygiene/biosecurity implemented, the clinical signs of enteritis are often observed. In such situations, bacteria and parasites are most often investigated microbiological agents. The aim of the study was to examine the prevalence of four enteric viruses, namely astrovirus (AstV), coronavirus (CoV), rotavirus (RoV) and parvovirus (PV) in Polish meat-type turkey flocks. Recently statistical methods have been used more often to explain which factors contribute to disease characteristics, thus we also attempted to determine whether a statistical correlation occurs between the presence of the viruses studied and the health condition and age of turkeys.

## Methods

### Sample collection

Between 2008 and 2011, a total of 2070 fecal swabs were collected from 207 turkey flocks (10 individual fecal swabs per flock) located in two regions of Poland (north-eastern, and western part). A lot of turkey farms, which account for about 70% of total production in Poland are located in these regions (Warmia-Mazury, Wielkopolska and Lubuskie voivodeships) [5]. The samples originated from both healthy and enteric turkeys, aged 1 to 140 days. To standardize sample collection, as well as to acquire additional information about flocks, turkey farm owners or veterinarians therein were provided with instructions for samples collection and questionnaires inquiring about such data as flock location, age of birds, number of birds in the flock, state of health, possible treatments. All samples were stored in -20 °C until processing.

The information about the health status of studied turkeys was obtained for 187 flocks. The health status was a subjective criterion, assessed in the descriptive form by individual veterinarians taking care of the studied turkey flocks. Based on this description the flocks were divided into three categories, i.e. healthy when the behavior and weight gain of birds were normal; PEC when any symptoms of enteritis, such as prolonged diarrhorea, low body gain and feed intake, or uneven growth occurred; and PEMS when more serious clinical signs of disease, including increased mortality, were observed.

The information about birds age was obtained for 149 flocks. Depending on the age, turkey flocks were separated into three following groups reflecting the production cycles: the growing phase of 1 to 4-week-old, the fattening phase of 5 to 12-week-old and the finishing phase of birds older than 13 weeks.

### Sample processing

After slow thawing, each individual swab (of 10 swabs sampled from flock) was hydrated in phosphate-buffered saline supplemented with antibiotics (100 U of penicillin with 100 mg of streptomycin/ml), incubated for 1 h at room temperature and clarified by centrifugation at 1500 g for 20 min. Five such obtained supernatants were combined into one pool so that two pooled samples were then tested per flock. Total RNA and DNA were then extracted from 250 μl of each two pools into 50 μl RNase-free water using commercial kits (RNeasy and DNeasy Mini Kits, respectively, Qiagen, Germany) according to manufacturer’s instruction. Extracted nucleic acids were stored in -70 °C for further molecular analysis.

### Molecular methods for enteric viruses

Four different assays were used for detection of astrovirus, coronavirus, parvovirus and rotavirus targeting the conservative regions of their genomes. In spite these viruses were detected both in diseased and healthy birds, they were recently regarded as those which are mainly responsible for enteritis in turkeys. Presence of astroviruses, irrespective of their affiliation to virus type was identified by using RT-PCR aimed at RNA dependent RNA polymerase gene fragment according to the protocol described by Tang et al. [6]. This method enables detection of both turkey astrovirus type 1 (TAstV-1) and type 2 (TAstV-2) as well as avian nephritis virus (ANV). Detection of CoV was performed in real-time RT-PCR aimed at 5′ untranslated region (5’UTR) fragment present in gammacoronaviruses of poultry i.e. turkey coronavirus and infectious bronchitis virus [7]. For parvovirus detection, primers which enable the amplification of non structural 1 (NS1) of *Aveparvovirus* gene were used [8]. For RoV identification the method according to Day et al. was applied [9]. This method enable the amplification of highly conserved non-structural protein 4 (NSP4) gene region which was believed not group specific. But next studies revealed that NSP4 gene of avian D, F and G rotavirus groups are only distantly related to that of avian group A so it seems that applied method is useful only for this group of rotavirus [10]. Details on primers sequences, annealing temperatures and sizes of expected amplicons are presented in the Table 1. Tests were performed using Qiagen One-Step RT-PCR kit (Qiagen, Germany) for RT-PCR, OptiTaq DNA Polymerase Kit (EURx, Poland) for PCR and QuantiTect Probe RT-PCR Kit (Qiagen, Germany) for real time RT-PCR. The above tests were conducted in a 2720 Thermal Cycler or 7500 Real Time PCR System for conventional or real time amplifications, respectively (both Applied Biosystems, USA). The details on reaction mixtures and the protocols of applied assays are available on request.

**Table 1:** Primers and probe used in the study

Primer name	Target virus	Target gene	Sequence	Annealing temperature (°C)	Amplicon (bp)	Ref
TAPG-L1	AstV	ORF1b	TGGTGGTGYTTYCTCAARA	50	601	[6]
TAPG-R1			GYCKGTCATCMCCRTARCA			
F30	RoV	NSP4	GGGCGTGCGGAAAGATGGAGAAC	50	630	[9]
R660			GGGGTTGGGGTACCAGGGATTAA			
PVF-1	PV	NS1	TTCTAATAACGATATCACTCAAGTTTC	55	500	[8]
PVR-1			TTTGCGCTTGCGGTGAAGTCTGGCTCG			
GU391			GCTTTTGAGCCTAGCGTT			
GL533	CoV	5’UTR	GCCATGTTGTCACTGTCTATTG	60	143	[7]
G probe			CACCACCAGAACCTGTCACCTC			

### Statistical analysis

The data obtained from completed questionnaires were analyzed. To assess the normal distribution, categorized graphs of normality and the Shapiro-Wilk test were used. To compare the quantitative correlation between age, health status as well as the number of identified viruses in all groups of birds, the Kruskal-Wallis test was used. In turn, the Mann-Whitney test was used to correlate the age of infected and uninfected birds. The interactions between qualitative variables, i.e. the age (categorized in groups) or health condition of the tested flocks and the prevalence of the studied viruses, as well as correlations between the incidences of the investigated pathogens, were also examined. These variables were summarized in the contingency tables which were examined by the independence chi-square (χ^2^) statistical test. The statistical methods which inform about the strength of the relationship between qualitative variables were then applied. Consequently, we calculated the Yule coefficients. However, these tests do not describe the nature of the relationships, and thus we applied the correspondence analysis (CA) to this end. The CA is the method of displaying the relationships between categorical variables as scatterplot diagrams; they are, however, not shown here, and only the conclusions from the obtained diagrams are discussed. We also implemented logistic regression (LR) model, which is also helpful in description of the strength and nature of the relationship between the variables. In the LR model, a dichotomous scale is used, so the health status of flocks has been rearranged as healthy or sick (PEC/PEMS) animals. Odds ratio (OR) and 95% confidence interval (CI) were calculated and the differences with *P* < 0.05 were considered as significant. Data analysis was performed using STATISTICA ver. 10 (StatSoft, Inc., 2011Dell Software, Aliso Viejo, CA, USA).

### Ethics statement

Sampling was conducted under the permission of the owners or other responsible persons. The birds in the farms were under the supervision of veterinarians, who took different samples as part of their routine work (i.e. as screening flocks for efficacy evaluation of applied vaccines or presence of any infections) and thus part of them were used in this study. For this reason, sampling did not require the approval of the Ethics Committee.

## Results

### Enteric viruses occurrence

Out of the 207 flocks tested, 137 (66.2%, 95% CI: 59.3-72.6) were positive for one or more enteric viruses. Among the positive flocks, 74 (54%, 95% CI: 45.3-62.6) were infected with one virus, 54 (39.4%, 95% CI: 31.2-48.1) with two viruses, and 9 (6.6%, 95% CI: 3.1-12.1) with three viruses. No flock was simultaneously infected with four studied viruses. AstV was the most commonly detected virus in the samples and its prevalence was 44.9% (95% CI: 38.0-52.0), followed by 27.5% prevalence of parvovirus (95% CI: 21.6-34.2), 18.8% of rotavirus (95% CI: 13.8-24.8), and 9.7% occurrence of coronavirus (95% CI: 6.0-14.5), as presented in Table 2.

**Table 2:** Frequencies of individual and multiple enteric virus infections detected in swab samples from all 207 commercial turkey flocks collected between 2008 and 2011

Item	TAstV	TCoV	RoV	TuPV
Number of viruses detected				
1 virus	39	7	7	21
2 viruses	45	9	26	28
3 viruses	9	4	6	8
Number of positive samples (%)	93 (44.9)	20 (9.7)	39 (18.8)	57 (27.5)

The majority of the samples were collected in 2008 (from 80 flocks) and the least flocks were sampled in 2010 (32 flocks). The prevalence of viruses varied depending on the year of the study. Generally, the frequencies of astrovirus, rotavirus and parvovirus occurrence during the four-year study revealed an increasing tendency. The most visible increase was observed in the case of RoV the occurrence of this virus was 12.5% in 2008 and rose up to 34.2% in 2011. Adversely, the occurrence of TCoV initially increased from 5% in 2008 up to 33.3% in 2010 and then decreased to a zero level of virus detection in 2011.

### Health status

Birds in 45 flocks were described as healthy, whereas in 94 flocks PEC and in 48 flocks PEMS symptoms were observed. The information on the health status of turkeys in 20 flocks was not available. Of the 45 flocks that were in good health condition, 25 were negative, 11 were positive for one virus and 9 were infected with two or three viruses. The most frequent viruses detected among healthy flocks was astrovirus (28.9%) and parvovirus (26.7%). RoV and TCoV were detected only in 4.4% and 6.7% of flocks without any disease signs, respectively. The frequencies of astrovirus, parvovirus, rotavirus and coronavirus detection in enteric birds were 44.9%, 27.5%, 18.8% and 9.7%, respectively. In 40 flocks showing clinical signs of enteritis no virus was detected. However, among the rest 102 diseased flocks 56.9% were infected with single virus. The most frequently identified in this group was AstV present in 51.7% of singly infected flocks. Thirty percent of flocks with enteritis were positive for more than one virus. Among enteric flocks co-infected with multiple viruses 50.8% included RoV. The results regarding correlations between virus prevalence and the health status of the flock are shown in Table 3.

**Table 3:** Distribution of viruses in studied samples from 187 commercial turkey flocks depending on health status of birds (healthy/PEC/PEMS)

Virus	Healthy (%)	PEC (%)	PEMS (%)	Total (%)
TAstV	6 (3.2)	18 (9.6)	12 (6.4)	36 (19.3)
TCoV	1 (0.5)	5 (2.7)	1 (0.5)	7 (3.7)
RoV	0	5 (2.7)	0	5 (2.7)
TuPV	4 (2.2)	10 (5.3)	7 (3.8)	21 (11.2)
Total one virus	11 (5.9)	38 (20.3)	20 (10.7)	69 (36.9)
Two viruses	8 (4.3)	24 (12.8)	16 (8.6)	48 (25.7)
Three viruses	1 (0.5)	3 (1.6)	1 (0.5)	5 (2.7)
Total coinfections	9 (4.8)	27 (14.4)	17 (9.1)	53 (28.3)
No virus	25 (13.4)	29 (15.5)	11 (5.9)	65 (34.8)
Total	45 (24.1)	94 (50.3)	48 (25.7)	187

### Flock age

Seventy flocks consisted of birds of 1 to 4 weeks age group (growing phase), 63 flocks comprised birds of 5 to 12 weeks age group (fattening phase), and the least numerous age group of over 13 weeks old (finishing phase) constituted 16 flocks. Table 4 presents the data about the numbers of diseased or healthy flocks and detected viruses according to the age of the flocks. In the case of 49 flocks, the information about the age of turkeys was not provided. Only 9 samples (12.9%) collected from turkeys in the growing phase came from flocks in good health condition although seven of them were infected with 1 or 2 viruses (Table 5). All the remaining samples originated from flocks that showed PEC or PEMS symptoms and 82% of them harbored 1 to 3 viruses per flock. The viruses most frequently detected in diseased turkeys in growing phase were TAstV (82%), RoV (38%) and PV (34%). Among 11 (18%) enteric flocks none of the 4 studied viruses were found.

**Table 4:** The numbers of diseased (PEC/PEMS) or healthy flocks and associated viruses according to the age of the flocks

Age	Flocks No.	PEC	PEMS	healthy	TAstV	TCoV	RoV	PV
1 to 4 wks	70	38	23	9	50	4	19	20
5 to 12 wks	63	26	15	22	13	6	3	18
> = 13 wks	16	12	1	3	2	3	0	0

**Table 5:** Distribution of virus number in diseased (PEC/PEMS) or healthy flocks according to the age of the flocks

Age	Number of				
	viruses	flocks	healthy	PEC	PEMS
1 to 4 wks	0	13	2	8	3
	1	24	4	10	10
	2	31	3	19	9
	3	2	0	1	1
5 to 12 wks	0	32	16	10	6
	1	23	3	14	6
	2	7	2	2	3
	3	1	1	0	0
> = 13 wks	0	11	3	8	0
	1	5	0	4	1
	2	0	0	0	0
	3	0	0	0	0

No enteric viruses were detected in either 16 flocks of fattening turkeys which showed clinical signs of intestinal disease or in 16 healthy flocks. In the remaining 6 healthy flocks one or more viruses were detected. Among 25 enteric flocks most of the samples (80%) were infected with one virus and two viruses were only present in 5 flocks. The PV (56%) and TAstV (36%) were the most frequently detected virus families in the samples from fattening flocks.

As regards turkeys in the finishing phase, most of them (8/16) showed mild clinical signs of enteritis independently of infection with any of the studied viruses. Among the remaining 5 diseased turkey flocks, three were infected with CoV and two with AstV.

### Statistical analysis

Only statistically significant results are presented; insignificant relationships of variables are not included in this section. Since the normality was not fulfilled, non-parametric Kruskal-Wallis and Mann-Whitney tests were selected to assess the differences in the average age between groups of healthy, PEC and PEMS birds. Additionally, due to the relatively restricted amount of data (especially for multifactorial models), two separate logistic regression (LR) models were used. The first model investigated the impact of virus infections on the variable ‘health condition’, while the second model explored the impact of the number of viruses and turkey’s age on the same variable, i.e. ‘health condition’.

#### Age and health status of turkeys

The results of applied statistical analysis revealed the existence of significant differences between the age of birds depending on their health status (healthy, PEC and PEMS). The Kruskal-Wallis test showed that the average age of healthy, PEC and PEMS turkeys differs significantly (*P* = 0.036). Multiple comparison test showed that this difference was only between healthy and PEMS turkeys (*P* = 0.03); healthy turkeys were about 7 weeks old and PEMS about 4 weeks old. Generally the older the turkeys were, the healthier they were. Such correlation was also implied by the independence chi-square test (*P* = 0.007, φ = 0.31). The calculated ORs of =3.57 and 3.75 indicated that the chance of PEC and PEMS symptoms in turkeys aged 1-4 weeks are above 3.5 times higher than the chance of such disease symptoms in the older group of 5-12-week-old animals. The OR =6.92 indicates that the possibility of PEMS relative to PEC symptoms in turkeys in the fattening phase is almost sevenfold higher than in turkeys over 13 weeks of age.

#### Age and astrovirus infection

The correlation between the age of turkeys and AstV infection was established by three statistical methods. The results of the Mann-Whitney test revealed that the difference between the age of uninfected and astrovirus-infected birds was significant (*P* = 0.0000). The median age of turkeys infected with AstVs was 3 weeks and uninfected turkeys were about 6 weeks old. The graphs of correspondence analysis also indicated relatively more astrovirus infections among the youngest individuals aged 1-4 weeks. The CA diagrams confirmed the correlation between the age and AstV infections. Such correlations were additionally confirmed in the independence chi-square test (*P* = 0.0000, φ = 0.50). The lowest prevalence of AstVs was in turkeys older than 13 weeks (12%, 95% CI: 1.5-36.4), slightly higher in turkeys aged 5-12 weeks (21%; 95% CI: 11.5-32.7), and the highest in young individuals aged 1-4 weeks (68%, 95% CI: 56.4%-78.1%). The calculated OR =0.12 indicates that the chance of astrovirus infection among turkeys aged 5-12 weeks is above eightfold lower than the chance of this infection in the youngest group of 1-4-week-olds. Furthermore, it is almost twofold lower (OR =0.51) in the oldest group (> = 13 weeks) than among turkeys aged 5-12 weeks.

#### Age and parvovirus infection

The correlation between turkey age and PV infection was only found by the independence chi-square statistical test (*P* = 0.04, φ = 0.20). No parvovirus infection was observed in turkeys older than 13 weeks while its prevalence in turkeys aged 5-12 weeks and 1-4 weeks was similar (29%, 95% CI:17.9-41.4 and 28%, 95% CI: 18.6-39.5, respectively). The CA diagrams confirmed this observation; the finishing group corresponded with PV-free birds while the growing and fattening phase groups corresponded with the infection.

#### Age and rotavirus infection

The correlation between the age of turkeys and RoV infection was also found by three applied methods. The results of the Mann-Whitney test revealed that the difference between the age of uninfected and rotavirus-infected birds was significant (*P* = 0.0000). The median age of turkeys infected with rotaviruses was 2 weeks and uninfected turkeys were about 5 weeks old. The independence chi-square test indicated a significant correlation between turkey age and RoV infection (*P* = 0.00007, φ = 0.35). There were no rotavirus infections among turkeys older than 13 weeks. The prevalence of RoVs in turkeys aged 5-12 weeks was about 5% (95% CI: 1.0-13.3) and in birds aged 1-4 weeks it reached the highest level (29%, 95% CI: 19.7-40.9), which means that the incidence of rotavirus infection diminished with age. The calculated OR =0.12 indicates that the chance of RoV infection among turkeys aged 5-12 weeks is above eightfold lower than the chance of this infection in the youngest group of 1-4-week-olds. The CA graphs confirmed this relationship.

#### Health status and rotavirus infection

The independence chi-square test revealed a significant relationship between RoV occurrence and the health status of turkeys (*P* = 0.01, φ = 0.22). The OR =6.96 indicates that the odds of disease symptoms in turkeys infected with rotaviruses are almost sevenfold higher than the odds of occurrence of these symptoms in the uninfected group. The CA plots also revealed that rotavirus infection is most strongly correlated with PEC symptoms in turkeys, and PEMS symptoms as well as healthy status correspond with turkeys uninfected with rotaviruses. The first LR model showed that only RoV infection has a significant impact on health status (diseased turkeys), with *P* = 0.017. The OR =5.06 indicates that the odds of disease symptoms of turkeys infected with rotaviruses are over fivefold higher than the odds of occurrence of these symptoms in the uninfected turkeys.

#### Age and number of viruses

The correlation between turkey age and the number of viruses was found in the Kruskal-Wallis test (*P* = 0.0000). Generally, the older the birds, the number of infecting viruses decreases. The median age of turkeys infected with one virus was 5 weeks, those infected with 2 viruses - 3 weeks, and in the case of birds uninfected with any virus it was 7 weeks- the differences between the age of differently infected birds were significant. Such correlation was also found in the independence chi-square test (*P* = 0.0000, φ = 0.49). The OR =2.58 indicates that the odds of infection with one virus in turkeys up to 4 weeks of age are more than 2.5-fold higher than the odds for the occurrence of such infections in turkeys aged 5 to 12 weeks and 1.7-fold higher in 5 to 12-week-old turkeys than in the oldest ones (≥13 weeks) (OR =1.73). Furthermore, the OR =4.3 indicates that the odds of infection with two viruses (as opposed to infection with one virus) in turkeys up to 4 weeks of age is more than fourfold higher than the odds for the occurrence of such infections at the age of 5 to 12 weeks. The CA plots also suggested the lowest number of infecting viruses/no infection in the oldest group, and the highest (2-3 viruses) in the youngest group, whereas turkeys aged 5 to 12 weeks were mostly associated with single virus infection.

#### Health status and number of viruses

The relationship between co-infection with two or three viruses and the health status of turkeys was identified in χ^2^ statistics (*P* = 0.046, φ = 0.26). The health condition of turkeys deteriorated with an increasing number of viruses; the OR =2.98 indicates that the odds of PEC symptoms in turkeys infected with one virus are almost threefold higher (and above fourfold higher for PEMS symptoms; OR =4.13) than the odds of occurrence of these symptoms in the uninfected turkeys. Additionally, the odds of PEMS symptoms (in relation to PEC symptoms) in turkeys infected with two viruses are about 1.27-fold higher than the odds of occurrence of these symptoms in birds infected with one virus and they are about 1.39-fold higher in turkeys infected with one virus than in the uninfected turkeys. Similarly, the CA diagrams also indicated that healthy turkeys had no virus infection, and infections with two or three different viruses most commonly resulted in disease symptoms. The second logistic regression model also indicated that the odds of disease symptoms in turkeys infected with one virus are more than 3.5-fold higher than in turkeys free from these infections (OR =3.61, *P* = 0.01) and that younger age of turkeys is a factor stimulating illness. The OR =2.75 (*P* = 0.04) indicated that the odds of PEC or PEMS symptoms in turkeys up to 4 weeks of life are more than 2.5-fold higher than in turkeys 5 to 12 weeks of age.

#### Correlations between investigated pathogens

The χ^2^ statistics revealed that there is a significant relationship between RoV and AstV infections (*P* = 0.0002; φ = 0.26). Among turkeys infected with rotavirus, about 72% were also infected with astroviruses. Contrastingly, only about 30% of AstV-infected birds were concomitantly infected with RoVs. However, among rotavirus-free turkeys, above 60% were also astrovirus-free and inversely, among AstV-free birds about 90% were also RoV-free. The OR =4.03 indicates that the odds of astrovirus (rotavirus) infection in turkeys infected with rotavirus (astrovirus) are about fourfold higher than the odds of occurrence of this infection in turkeys uninfected with rotavirus (astrovirus).

## Discussion

The present study was undertaken to estimate the prevalence of four enteric viruses, namely astrovirus, coronavirus, parvovirus and rotavirus in commercial meat-type turkey farms in Poland. As the role of these viruses in the development of enteritis is still unclear, statistical analysis was also applied in an attempt to better understand the influence of these viruses on the health status of studied turkey flocks.

All applied statistical analyses confirmed the common knowledge that the older the turkeys are, the healthier they are. The intestines during the first weeks of bird’s life (up to 2-3 week) are in a phase of morphological, biochemical, immunological and molecular maturation and during this time they are concomitantly exposed to a variety of factors [11]. They contain their own microbial communities which are initially influenced by the breeder, but later also by environment [12]. The health status of intestines depends on maintaining the balance of all components of the turkey gut and environmental factors. Any disturbances in this balance may cause enteritis. Clinical signs of enteritis in turkey aged 1-4 weeks are most probably due to the disturbance of this delicate balance between various factors in immature intestines.

Enteric viruses were detected in 66.2% of Polish commercial turkey flocks. Among the studied viruses, the most frequently detected in both healthy as well as in enteric turkeys was AstV, accounting for 28.9% and 47.2%, respectively. Our previous studies on Polish turkey flocks also revealed the presence of turkey astroviruses, independently of detected types (TAstV-1, TAstV-2 or ANV), in both turkeys experiencing enteritis as well as in clinically healthy birds [13]. Previously applied statistical methods indicated a weak correlation between astrovirus incidence and the health status, with calculated coefficients between combined TAstV-1 and TAstV-2 presence or TAstV-2 presence and the health status equal to 0.14 and 0.1, respectively [13]. However, rank Spearman correlation used at that time seems to be not fully appropriate for qualitative variables. Thus in this study we have applied other methods, more useful for such kind of analysis. None of them indicated any correlation between AstVs prevalence and turkey health status. There are many reports of astrovirus presence in turkeys, with the prevalence varying from about 24 to 100% depending on the methods used for virus detection and the country; however, according to our knowledge, none of them specifically explored the impact of astroviruses on the health of turkeys. The surveys of commercial turkeys conducted in the USA revealed that 47.2 and 100% of the analyzed samples were astrovirus-positive [14]. The majority of detected astroviruses belonged to TAstV-2 and their level ranged from 69.6 to 71.8%. The occurrence of TAstV-1 and ANV was less frequent, accounting for 9.8-28.1% and 2.7 – 12.5%, respectively, but all of these turkey astrovirus types were detected irrespectively of the turkey health status [14]. Brazilian studies also revealed a high prevalence of astroviruses in turkey flocks ranging from 44% to 81.6% [15, 16]. The share of individual astrovirus types was different than in the USA, but no clear influence on the health status was suggested. Cattoli et al. reported TAstV in Italian and Spanish turkey farms with enteritis [17]. On the other hand, Jindal et al. reported TAstV-2 in apparently healthy breeder turkey poults by RT-PCR; 47.2% of pooled fecal samples were positive [18]. Recently, TAstV-2, TAstV-1 and ANV were detected in 50%, 20% and 13.8% of market-age turkeys with lower weight but also in 33%, 20% and 12.5% of turkeys with standard breed character, respectively [3, 19]. Moreover, the same author found TAstV-2 in fecal swabs of both experimentally infected and PBS-inoculated turkeys [3]. In several reports the existence of two turkey astrovirus pathotypes were suspected: pathogenic and non-pathogenic [19, 20]. Presented results of applied statistical methods do not exclude the existence of such pathotypes, but it seems that the frequency of pathogenic astrovirus strains occurrence is rather low.

PVs were detected in 26.7% and 27.5% of healthy and enteric birds, respectively. Slightly higher rates of parvovirus infection in Polish turkey farms were demonstrated previously [21]. However, the occurrence of parvoviruses in turkey flocks in Poland was lower when compared to the 71-78% prevalence in commercial turkey flocks reported in a survey in the USA between 2003 and 2008 [8, 22]. The presence of PV infections in 46.9% of Hungarian turkey flocks experiencing enteric disease syndrome was also reported recently [23]. However, it should be noted, that most of the information about the PV came from studies of birds with enteritis problems. On the other hand, enteritis was experimentally reproduced in chickens infected with intestinal content containing parvoviral particles; however, the used inoculum might have contained other unknown viruses (apart from astrovirus, rotavirus and reovirus tested) which might have caused disease symptoms [24]. Our results identified the presence of PV with the same intensity in healthy individuals as in diseased ones. Moreover, the applied statistical investigation did not indicate any correlation between parvoviruses and turkey health status.

The overall observed frequency of RoV detection was 20.8%, but its prevalence was different in regards to health status of studied turkeys: in enteric flocks (21.1%) it was above fourfold higher than in healthy ones (4.7%). All three applied different statistical methods have indicated the correlation between rotavirus prevalence and turkey health status. Calculated odds of disease symptoms in turkeys infected with RoV, depending on the methods used, were from five- to almost sevenfold higher than the occurrence of these symptoms in the uninfected group. Rotaviruses are a major cause of acute enteritis in young children and in several mammalian and avian species [25–27]. However, the impact of RoV on the health of turkeys is ambiguous, as its presence was reported in healthy as well as in enteric birds. Periodic monitoring of eight turkey flocks in North Carolina revealed the presence of rotaviruses in 69.7% of all tested samples during the whole production cycle. Moreover, RoVs were also detected in poults at the hatchery and vertical transmission was suggested as the reason of such detection. Interestingly, all studied flocks were described as healthy and normally performing by field personnel, but compared to the turkeys raised in experimental conditions they had significantly lower body weight and worse feed conversion rate [1]. The monitoring of enteric turkeys in Minnesota showed the presence of RoV in 48.3-93% of tested flocks [28, 29]. In subsequent studies by the above authors, the monitoring of five flocks of apparently healthy breeder turkeys over the period of 9 weeks revealed RoV presence with maximum occurrence until 5 weeks of age [18]. In a Brazilian study, rotavirus infections were identified in 52.6% of surveyed turkey farms; its prevalence in enteric and healthy flocks was 57.8% and 39.3%, respectively [16]. The presence of RoV in 18.8% of Polish diseased and healthy turkey flocks has also been previously reported [30]. It should be stressed that in all abovementioned studies the same protocol was used for RV detection as in present study [9]. The possible reason for the observed ambiguous impact of rotavirus infection on enteritis is the circulation of virus strains that differ in pathogenicity. Although our previous studies reported only several amino acid changes in NSP4 both in strains identified in enteric and healthy turkeys, the biological characteristics of RoVs, such as the replication ability, virulence and pathogenicity, are determined by a combination of many genes, such as VP4, VP7, VP3, NSP1, NSP2, and NSP4 gene [31]. Thus in order to get more insight into the pathogenicity as well as to establish a definite classification of detected rotavirus strain, the complete genome sequence should be characterized.

Turkey coronaviruses were found in 9.7% of the Polish flocks analyzed between 2008 and 2010. They were detected in 6.7% of healthy flocks and 9.2% of flocks with clinical signs of enteritis. Generally, TCoV is directly connected with enteritis [4]. The virus was responsible for enormous losses in turkey production in Minnesota in the early 1970s and then for several outbreaks in multiple states in the United States since the 1990s [32, 33]. Maurel et al. reported that 37% of intestinal samples from diseased turkey flocks in France were TCoV-positive [34]. Villareal et al. demonstrated the presence of TCoV in 82.4% of studied diseased turkey flocks, but also in one apparently normal [15]. In another study from Brazil, TCoVs were detected in 71.1% and 28.6% of enteric and healthy flocks, respectively [16]. The lack of clinical signs of enteritis in spite of detected infection with turkey coronavirus may be interpreted in a few ways. Firstly, the sampling time – compilation of the results when the sampling was performed in the early stages of infection before the onset of clinical symptoms of the disease or at a later stage when clinical symptoms can be observed in the presence of the virus below its detection limit will lead to doubtfulness. Secondly, the existence of turkey coronaviruses with various pathogenicity. In addition, the severity of the disease could be influenced by other zoohygenic or infectious factors.

The results of most of the applied statistical methods have indicated clear correlations between astrovirus and rotavirus incidences, and this corresponds with the results of metagenomic studies which revealed that the most abundant RNA virus families in the gut of clinically normal 5-week old turkeys were *Astroviridae* and *Reoviridae*, and of these families, viruses of *Rotavirus* and *Astrovirus* genera [35]. We also found the correlation between AstV and RoV and the age of turkeys in studied flocks pointing at young birds as more susceptible to infection with these viruses. Our observations statistically support the view that astroviruses and rotaviruses are rarely detected after 4 weeks or below 6 weeks of age, respectively [36].

## Conclusions

Our results revealed the prevalence of astroviruses, coronaviruses, rotaviruses and parvoviruses in Polish turkey flocks. The presence of detected viruses was commonly identified in flocks of both clinically normal and enteric birds. Moreover, we have tried to statistically analyze the obtained results for any correlations between such variables as health status/virus species/number of detected viruses/age groups. The dependence between rotavirus infection and the health status of turkeys was found. The occurrence of TAstV, TuPV and TCoV had no statistical effect on the development of clinical signs of enteritis. Additionally, our results suggest that infection with two or three viruses also has an effect on the health of turkeys, which is in line with recent observations, so far statistically unsupported, suggesting that viral co-infections may be relevant in the onset and the severity of enteric disease [16, 18]. Final determination of the role of these viruses could be clarified in experimental conditions using SPF or commercial turkeys. However, in order to do this, the virus should be available as propagated on embryos/cell lines, which however currently has not been achieved.

## Abbreviations

AstV: Astrovirus
CoV: Coronavirus
PV: Parvovirus
RoV: Rotavirus
